# Psychometric properties of the Chinese version of procrastination assessment scale for students

**DOI:** 10.3389/fpsyg.2022.1016116

**Published:** 2022-10-06

**Authors:** Michael C. W. Yip, Olive L. L. Chung

**Affiliations:** Department of Psychology, The Education University of Hong Kong, Hong Kong, Hong Kong SAR, China

**Keywords:** academic procrastination, psychometric properties, Chinese version, PASS, university students

## Abstract

The procrastination assessment scale for students (PASS) has been used widely in evaluating the patterns of university students’ procrastination on academic tasks and their procrastination behavior. The present study validated the psychometric properties of a Chinese version of the PASS (PASS-C) by recruiting two representative independent sample of Hong Kong Chinese university students (S1 used in the EFA study: 506; S2 used in the CFA study: 506). The results confirmed that this modified Chinese version is a valid and appropriate tool to assess university students’ procrastination tendencies in Chinese educational settings.

## Introduction

Academic procrastination is the intentional action of unnecessarily delaying academic tasks persistently until it reaches a level that induces subjective discomfort (Solomon and Rothblum, 1984). This is a complex phenomenon that involves different cognitive, affective, and behavioral components (Rothblum et al., 1986). Dating back to the earlier literature and from the estimations made by Ellis and Knaus (1977), 95% of American college students had experienced academic procrastination. Similar research findings also found that 25% of Caucasian-American college students had problems with procrastinating on different academic tasks (Solomon and Rothblum, 1984) and 30–45% of African American college students had the same problems (Clark and Hill, 1994). The magnitude of academic procrastination has also been observed to be related negatively to academic performance (GPA) because of the poor performance in different academic tasks, such as missing submission deadlines for assignments or writing up term essays (Semb et al., 1979; Solomon and Rothblum, 1984; Beswick et al., 1988). The trend of academic procrastination is continued as a serious concern in the educational sector, particularly in the pandemic situation (Holzer et al., 2021; Hong J. C. et al., 2021; Melgaard et al., 2022).

Academic procrastination has been shown to be related to some important variables, such as students’ learning motivation and learning strategies (Dewitte and Lens, 2000; Ferrari and Scher, 2000; Dewitte and Schouwenburg, 2002; Klingsieck et al., 2013). In particular, past studies have found that self-efficacy, learning motivation, effort regulation, perfectionism (fear of failure) and time management are all reflective characteristics of academic procrastination (Van Eerde, 2003; Howell and Watson, 2007; Klassen et al., 2008; Rakes and Dunn, 2010; Closson and Boutilier, 2017; Wolters et al., 2017); and these factors are all negatively related to academic performance. For example, Christopher (1998) reported that intrinsically motivated students usually procrastinated less than their externally motivated peers; hence suggesting that a lack of learning motivation contributes to more procrastination behaviors (Senecal et al., 1995; Steel, 2007). Similarity, in their study, Zarrin et al. (2020) reported that fear of failure (positively related) and self-regulation (negatively related) were associated with academic procrastination, and thus influencing the learning performance of students. Conclusively, this line of research argues that academic procrastination is a reliable predictor of poor academic performance mediated by the learning strategies (Van Eerde, 2003; Goda et al., 2015). All the aforementioned variables are related closely to students’ learning strategies and those learning strategies are in turns related to the learners’ cultural backgrounds (Liem and Bernardo, 2013; Yip, 2017). An earlier study conducted by (Kember and Gow, 1990) concluded that different cultural values occur in different educational systems used in different places (Lau, 1992; Purdie and Hattie, 1996; Purdie et al., 1996; Yamauchi and Tanaka, 1998; Salili et al., 2001b; Hau and Ho, 2010). For example, almost all the Chinese and Asian students in these studies (or Canadian Chinese students in Salili et al., 2001a study) shared the common belief that their parents and families have high expectations of them (*cf*. Fu and Markus, 2014). This strong belief became a robust motivator for these students in many respects, including learning and studying in school (Salili, 1996; Kember and Watkins, 2010; Suzuki and Sun, 2017; Ng and Wang, 2019). A recent study by Yip (2021) also drew a similar conclusion about Japanese students’ view of learning. These types of cultural and societal differences between western and East Asian countries induce not only different views of learning and learning motivation (see Liem and Bernardo, 2013; Schunk and Greene, 2017; Yip, 2017; King, 2018 for reviews) but also learning-related habits, such as academic procrastination (Steel, 2007; Batool, 2020).

Empirical investigations of academic procrastination have come largely from the Western literature so far. Only a few studies have focused on the same issue in Asian countries and Chinese-speaking groups (Tan et al., 2008; Yao, 2009; Shih, 2017; Zhao et al., 2021). In a recent report, Chen et al. (2022) observed that “Concern over mistakes (a dimension of perfectionism)” of students was positively associated with academic procrastination, and this high personal standard of learning was linked up with the parental expectation, and hence the magnitude of the same factor was relatively lower than their western counterpart. The research group of Klassen et al. (2009) also found that Singaporean adolescents reported higher levels of procrastination and lower levels of self-efficacy for self-regulation than Canadian adolescents in the multigroup SEM analysis. Similar pattern of cross-cultural difference on learning motivation also occurred in their follow up study (Klassen et al., 2010).

Therefore, to study the prevalence of students’ academic procrastination and to understand their procrastinating behaviors in Asian countries (and Chinese-speaking regions in particular), it is necessary to have a valid instrument that is appropriate for the Chinese culture, and hence this was the main objective of the present study.

There are many different inventories in the literature, but the procrastination assessment scale for students (PASS) constructed by Solomon and Rothblum (1984) has been one of the most widely used pioneering classical inventories to investigate academic procrastination during the last three decades (Ferrari et al., 1995; Bridges and Roig, 1997; Alexander and Onwuegbuzie, 2007; Burnam et al., 2014; Abdollahi et al., 2020; Batool, 2020; Suárez-Perdomo et al., 2022). This was developed specifically to assess the prevalence of students’ academic procrastination in western cultures. The inventory is a self-reported questionnaire that records the frequencies and reasons for students’ academic procrastination. It has been used in many psychological and educational studies and has demonstrated that students’ academic procrastination was highly correlated with their different kinds of behavioral, cognitive, and affective behaviors (Solomon and Rothblum, 1984), personality trait dimensions (Johnson and Bloom, 1995; Schouwenberg and Lay, 1995; Watson, 2001), levels of anxiety (Lay and Silverman, 1996; Onwuegbuzie, 2004) and cognitive learning strategies (Rodarte-Luna and Sherry, 2008).

The original version of PASS consists of 26 items that attempted to assess the different possible reasons for students’ academic procrastination in specific scenarios (i.e., approaching due date of final term paper). Participants are asked to rate statements according to how much they reflect their reasons for procrastinating on a 5-point Likert scale (from “Not at all reflects why I procrastinated” to “Definitely reflects why I procrastinated”). Possible reasons are, for example, perfectionism, evaluation anxiety, low self-esteem, aversion of task, laziness, bad time management, difficulty in making decisions, peer influence, dependency, lack of assertion, risk taking, fear of success, and rebellion against control. This instrument can be used to form a core explanatory model of procrastination for university students in western cultures.

### Objective of the present study

In the present study, we examined the psychometric properties of a Chinese version of the PASS-C. Assuming the intrinsic differences and characteristics between Chinese and western cultures and educational systems (King et al., 2018), two independent samples (one for EFA and the other for CFA) were used. In order to validate the PASS-C, we conducted several statistical analyses to investigate the latent structure of PASS-C: [1] its underlying factor structure, and [2] the internal consistency coefficients of the scores based on the *n*-factor model extracted in [1]. We also [3] evaluated the criterion-related validity by examining correlations between the sub-scale (Fear of failure) of PASS-C and the Chinese version of Performance Failure Appraisal Inventory (PFAI-C) validated by Cho and Lu (2005). As well, we compared the PASS-C with the original PASS measure (Solomon and Rothblum, 1984).

## Materials and methods

### Participants

We recruited 1,028 university students [625 females (61%) and 403 males (39%) with a mean age of 23.1 (SD = 2.4)] randomly from eight different universities (including both publicly funded and private universities) in Hong Kong to participate in the present study. All participants were undergraduate students coming from different majors in their respective universities. They were all local Hong Kong Chinese. Altogether, there were 1,027 questionnaires returned, but 15 were discarded either due to incomplete answers (9 students) or because they were completed by foreign exchange students (6 students), and the final sample size was 1,012. Of these participants, 506 were assigned randomly as sample one (used in the EFA study) and the remaining 506 were assigned as sample two (used in the CFA study). All of them took part in the study voluntarily and they were informed verbally about the procedure of the study and gave their written, signed consent. This study was approved by the HREC of the Education University of Hong Kong and all methods and procedures were carried out according to the guidelines and regulations approved by the University.

### Measure

This Chinese version of PASS (PASS-C) was first translated by one of the authors (a bilingual psycholinguist) and then reviewed by both the co-author and the two authors’ their research assistants. During the translation process, an effort was made to ensure the appropriateness of the language and the cultural and educational context in order to maintain the content/face validity of the inventory. The Chinese version was again back translated into English by a professional bilingual editor. The resultant English back-translation version was further reviewed by the authors and the expert panel (including two educational psychologists). All the final modifications received unanimous agreement among the whole group (*cf.* Yip, 2013). Hence, the PASS-C (see the sample items in Appendix) consisted of the original 26 items (as in PASS), using a 5-point Likert scale (from “Never reflect why I procrastinated” to “Completely reflect why I procrastinated”) to reflect on the reasons why college students procrastinate.

In addition, the Chinese version of Performance Failure Appraisal Inventory, PFAI-C (Cho and Lu, 2005), a 25-item inventory using a 5-point scale, was used to assess the psychological construct “Fear of failure.” Here, we used the items to measure the factor of “Fear of failure” in the inventory to evaluate the criterion-validity of the PASS-C. It was hypothesized that both sub-scale scores would be positively correlated with the Chinese version of the Performance Failure Appraisal Inventory (PFAI-C) score.

### Procedure

Two research assistants distributed the two inventories in the libraries and canteens of the universities randomly. They invited each participant to complete the two questionnaires in about fifteen minutes. The two research assistants also reminded each participant that this was a simple self-report survey about their own procrastination tendencies and antecedents of academic procrastination. To ensure anonymity, the participants were not required to include their names.

## Results and discussion

Before we analyzed the data, the distribution of responses to the items of each sub-scale was calculated by examining skewness and kurtosis. The results showed that they were in normal distribution (skewness between −0.16 and −0.32 and the kurtosis was between 2.85 and 4.08). Moreover, we screened out the extreme values and then removed them from the data set for the subsequent analysis. In the determination of extreme values, all the scores were transformed to standardized z-scores. Scores that were beyond three standard deviations from the mean were considered as the extreme values. The extreme cases were rare (0.36%) in the whole dataset.

Following our analysis plan, we first explored the underlying factor structure of the PASS-C by conducting a varimax-rotated principal components analysis of the 506 participants’ scores (sample 1). As postulated in the original PASS, the inventory had seven factors, but six of these were extracted based on the present dataset (extracted as a factor if the eigenvalues above 1.5). The six-factor model accounted for 77.26% of the variance: Factor 1 (Fear of Failure – items 19, 24, 26, 33, 39, 40, and 42) 23.52%, Factor 2 (Task aversiveness – items 27, 28, 34, 35, and 43) 16.26%, Factor 3 (Difficulty in deciding – items 21, 23, and 31) 11.27%, Factor 4 (Dependency – items 20, 29, and 41) 10.71%, Factor 5 (Risk taking – items 30, 36) 10.28% and Factor 6 (Rebellion against control – items 25, 38) 5.22%. The pattern of structure coefficients of PASS-C are presented in Table 1.

**Table 1:** Factor structure of the modified Chinese version procrastination assessment scale for students (PASS-C) after varimax-rotation.

	Factors					
Item no.	I	II	III	IV	V	VI
19	0.02	0.07	0.03	***0.77***	0.05	0.09
24	0.12	0.08	0.07	***0.65***	0.06	0.08
26	0.05	0.11	0.08	***0.67***	−0.14	0.16
33	0.05	0.07	0.12	***0.64***	0.17	0.04
39	0.03	0.08	0.15	***0.70***	0.03	0.06
40	0.05	0.31	0.08	***0.61***	0.15	0.07
42	0.05	0.01	0.13	***0.67***	0.02	0.11
21	***0.68***	0.24	−0.03	0.09	0.04	−0.04
23	***0.64***	−0.15	0.07	0.07	0.06	0.05
31	***0.71***	0.22	0.06	0.16	0.18	0.16
27	0.06	0.03	−0.01	0.13	***0.58***	0.08
28	−0.07	0.05	0.04	0.07	***0.62***	0.02
34	0.05	0.11	−0.12	0.06	***0.62***	0.07
35	0.21	−0.02	0.09	0.02	***0.58***	0.01
43	0.14	0.08	0.07	−0.05	***0.67***	0.05
20	0.09	0.23	0.06	0.16	0.05	***0.58***
29	0.05	0.08	0.08	0.05	0.12	***0.66***
41	0.06	0.16	0.12	0.04	0.23	***0.76***
30	0.06	0.04	***0.71***	0.07	0.15	0.01
36	0.18	0.01	***0.62***	0.03	0.11	0.03
25	0.12	***0.61***	0.05	0.05	0.13	0.07
38	0.05	***0.69***	0.02	0.14	0.08	0.06
22	0.12	0.06	0.08	0.03	0.08	0.05
32	0.01	0.04	0.15	0.31	0.01	0.18
37	0.07	0.07	0.06	0.05	0.03	0.07
44	0.03	0.21	0.05	0.04	0.03	0.05

Factor loadings greater than 0.5 are in bolded italics.

**Figure 1:**
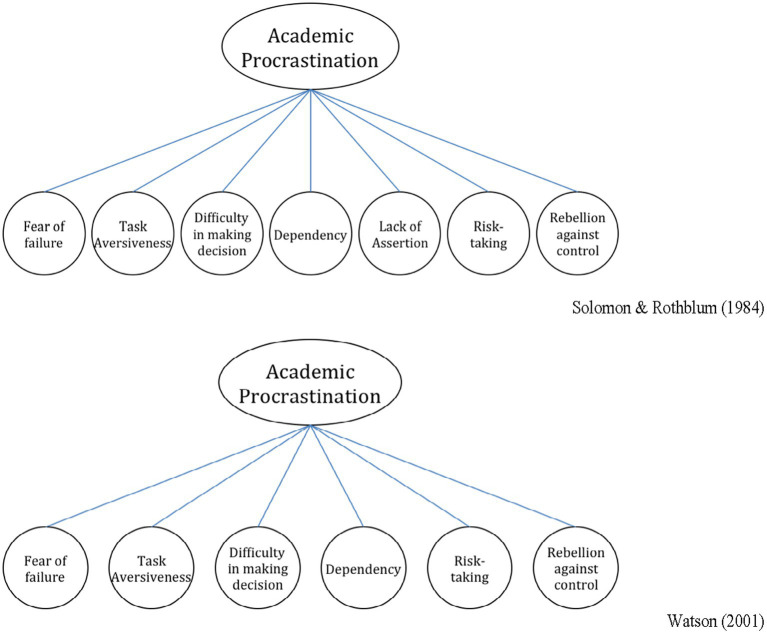
Comparison between PASS model of Solomon and Rothblum (1984) and Watson (2001).

Second, to assess the validity of the above-proposed measurement model (through principal components analysis) of PASS-C, confirmatory factor analyses (CFAs) were conducted to test the underlying factor structure (construct validity) of PASS-C based on the scores obtained from the other 506 participants (sample 2). The CFAs were conducted using IBM SPSS AMOS 24 and parameter estimates were generated using full information maximum likelihood (FIML) and tests of goodness of fit. Two competing models were tested: Model 1 (a seven-factor model) and Model 2 (a six-factor model). These were derived from the work of Solomon and Rothblum (1984) and Watson (2001) (see Figure 1). That is, aligned with the work of Solomon and Rothblum (1984), all items were hypothesized *a priori* that could be explained by seven higher-order factors in Model 1 or by six higher-order factors in Model 2 (Watson, 2001). In the present study, we evaluated the model fit using χ^2^ statistics, comparative fit index (CFI), non-normed-fit index (NNFI), root mean square error of approximation (RMSEA), and 90% confidence interval (CI) of RMSEA. CFI and NNFI value ≥0.9 and RMSEA value ≤0.08 were considered as indicators of good model-data fit (Bentler and Bonett, 1980; Browne and Cudeck, 1993; Hu and Bentler, 1999). Furthermore, for the criterion-related validity, we evaluated the scores by examining the correlation coefficients between the PASS-C and PFAI-C.

**Figure 2:**
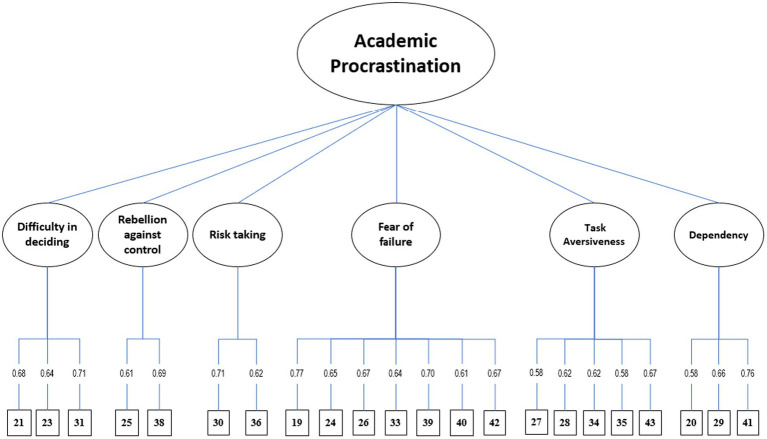
Model of Chinese version procastination assesment scale for student (PASS-C).

The fit indices for the two CFA models are presented in Table 2. Model 1 was not an acceptable fit for the present data (CFI and NNFI ≤0.9). Model 2 fitted the present dataset quite well in terms of the aforesaid criteria (except the RMSEA was at the critical margin to the criterion, 0.08). Therefore, Model 2 (Figure 2) was selected as the best fitted model to represent the underlying factor structure of PASS-C after removing four items [22, 32, 37, 44] from the overall model (due to the extremely low factor loadings and not statistically significant in EFA). The factor loadings of all the remaining 22 items for this model were statistically significant (*p* < 0.05). In addition, we also tested the one-factor model of all the 26 items loaded on a single latent factor. However, this model did not converge and another further test could not be conducted on this one-factor model. Such model mis-specification indicated a poor goodness of fit of this one-factor model with the current dataset.

**Table 2:** A summary of the CFA results of the two competing models.

Model	χ^2^	df	Value of *p*	CFI	NNFI	RMSEA	90% CI
Seven-factor hierarchical model	6295.8	192	<0.001	0.86	0.087	0.08	0.06, 0.09
2. Six-factor hierarchical model	5471.3	186	<0.001	0.92	0.093	0.08	0.06, 0.09

χ^2^, chi-square statistics; df, degree of freedom; CFI, comparative fit index; NNFI, non-normed fit index; RMSEA, root mean square error of approximation; CI, confidence interval.

Cronbach’s coefficients (α) for each factor (0.68–0.84) and the correlation matrix of PASS-C are presented in Tables 3, 4, respectively.

**Table 3:** Cronbach’s coefficients (α) for the Chinese version procrastination assessment scale for students (PASS-C).

Factors	No. of items	Cronbach’s α
Fear of failure	7	0.842
Task aversiveness	5	0.768
Difficulty in deciding	3	0.726
Dependency	3	0.691
Risk taking	2	0.706
Rebellion against control	2	0.684

**Table 4:** Inter-correlations of factors in PASS-C.

Factor	1	2	3	4	5
1					
2	0.45*				
3	0.36*	0.33*			
4	0.34*	0.42*	0.38*		
5	0.21	0.25*	0.41*	0.53*	
6	0.17	0.16	0.23*	0.49*	0.68*

^1^Factor 1, fear of failure; Factor 2, task aversiveness; factor 3, difficulty in deciding; factor 4, dependency; factor 5, risk taking; factor 6, rebellion against control.

^2^*p < 0.05.

Finally, to test criterion-validity, we examined the correlations between the score for the “Fear of failure” factor of the PASS-C and the PFAI-C. We found that there was a high and positive correlation between the two sub-scales, *r* = 0.78, *p* < 0.05. Therefore, the result demonstrated good convergent validity for PASS-C.

Overall, scores obtained through the large-scale survey confirmed that this Chinese version (PASS-C) was suitable to assess university students’ procrastination tendencies in Chinese educational settings because the present dataset revealed consistent patterns of scores in comparison to the PASS. All the coefficients for each sub-scale were within ±0.03, consistent with the alpha coefficients reported in the norm manual of PASS.

The indices generated from the CFA models indicted that the present results were slightly different from the seven-factor structure postulated in the PASS model (Solomon and Rothblum, 1984). Rather, a six-factor model seems to be more applicable to explain the present set of data (*cf.* Watson, 2001). Two items in the reduced factor “Lack of assertion” (item 23: ‘There’s some information you needed to ask the professor, but you felt uncomfortable approaching him/her.’ and item 29: ‘You had difficulty requesting information from other people.’) were loaded separately on two other different factors in the new six-factor model based on the good fit of the CFI and the NNFI index. The re-distribution of items is in line with the personal motivation and learning approaches of Asian students, in particular Chinese students, which are based on parental and societal expectations to achieve highly and hence not always the same as those of their western counterparts (Ho and Hau, 2008; Hau and Ho, 2010; Yip, 2017; Afzal and Jami, 2018; King et al., 2018).

Finally, there are two issues that need to be investigated further. One is that students’ self-reported scores for procrastination on academic tasks and the procrastination behavior could not reflect their internal cognitive processing in reliable or rigorous ways (e.g., lack of other behavioral or physiological indicators, Del Giudice, 2020). The second is that university students’ individual backgrounds may likely affect their procrastination behaviors (i.e., students in different years of their studies or studying different major subjects may be more likely than others to procrastinate academically). Hence, in our laboratory we are designing further studies (1) using both self-reported measures and other experimental tasks to produce a more comprehensive assessment of the relevant learning behaviors and (2) to carefully examine the patterns between procrastination behaviors and students’ individual backgrounds or other learner-related variables. Despite its limitations, this study is probably the first to validate the PASS and the present results provide reasonable support that the PASS-C could be seen as a reliable and useful instrument to be employed in different psycho-educational as well as counseling research projects that involve Chinese cultures (*cf.* Yao, 2009; Zhang et al., 2018; Hong W. et al., 2021).

## Author’s note

We thank Carol Chan, Claire Kan, Cathy Lee, Katherine Leung, Ken Liu and Chloe Shek for their excellent assistance in the present study as well as the constructive comments from the reviewers. Thanks also go to Professors Laura Solomon and Esther Rothblum for providing us useful information of the original PASS inventory.

## Data availability statement

The original contributions presented in the study are included in the article/supplementary material, further inquiries can be directed to the corresponding author.

## Ethics statement

The studies involving human participants were reviewed and approved by HREC of the Education University of Hong Kong. The patients/participants provided their written informed consent to participate in this study.

## Author contributions

All authors listed have made a substantial, direct, and intellectual contribution to the work and approved it for publication.

## Funding

This research was supported by a Departmental Research Grant (DRG#04160) from the Education University of Hong Kong.

## Conflict of interest

The authors declare that the research was conducted in the absence of any commercial or financial relationships that could be construed as a potential conflict of interest.

## Publisher’s note

All claims expressed in this article are solely those of the authors and do not necessarily represent those of their affiliated organizations, or those of the publisher, the editors and the reviewers. Any product that may be evaluated in this article, or claim that may be made by its manufacturer, is not guaranteed or endorsed by the publisher.
